# One-Pot Synthesis of Thiol-Modified Liquid Crystals Conjugated Fluorescent Gold Nanoclusters

**DOI:** 10.3390/nano10091755

**Published:** 2020-09-06

**Authors:** Po-Hsuan Hsu, Sibidou Yougbaré, Jui-Chi Kuo, Dyah Ika Krisnawati, Achmad Jazidie, Mohammad Nuh, Po-Ting Chou, Yu-Cheng Hsiao, Tsung-Rong Kuo

**Affiliations:** 1 School of Biomedical Engineering, College of Biomedical Engineering, Taipei Medical University, Taipei 11031, Taiwan; b812106028@tmu.edu.tw (P.-H.S.); b812105002@tmu.edu.tw (J.-C.K.); 2 International Ph.D. Program in Biomedical Engineering, College of Biomedical Engineering, Taipei Medical University, Taipei 11031, Taiwan; d845107003@tmu.edu.tw; 3 Institut de Recherche en Sciences de la Santé (IRSS-DRCO), 03 B.P 7192, Ouagadougou 03, Nanoro, Burkina Faso; 4 Dharma Husada Nursing Academy, Kediri, East Java 64114, Indonesia; dyahkrisna77@gmail.com; 5 Department of Electrical Engineering, Institut Teknologi Sepuluh Nopember, Surabaya 60111, Indonesia; rektor@unusa.ac.id; 6 Nahdlatul Ulama Surabaya University, Surabaya 60111, Indonesia; 7 Department of Biomedical Engineering, Institut Teknologi Sepuluh Nopember, Surabaya 60111, Indonesia; nuh@ee.its.ac.id or; 8 Graduate Institute of Biomedical Optomechatronics, Taipei Medical University, Taipei 11031, Taiwan; d97223108@ntu.edu.tw; 9 Graduate Institute of Nanomedicine and Medical Engineering, College of Biomedical Engineering, Taipei Medical University, Taipei 11031, Taiwan

**Keywords:** liquid crystals, thiolate ligand, gold nanoclusters, fluorescence, ligand–metal charge transfer, nanobiotechnology

## Abstract

Gold nanoclusters (AuNCs) and liquid crystals (LCs) have shown great potential in nanobiotechnology applications due to their unique optical and structural properties. Herein, the hardcore of the 4-cyano biphenyl group for commonly used LCs of 4-cyano-4′-pentylbiphenyl (5CB) was utilized to synthesize 4′-(2-mercaptoethyl)-(1,1′-biphenyl)-4-carbonitrile (TAT-12) based on Suzuki coupling and Appel reaction. The structural and optical properties of thiol-modified TAT-12 LCs were demonstrated by nuclear magnetic resonance (NMR) spectroscopy, ultraviolet-visible (UV-vis) spectroscopy and differential scanning calorimetry (DSC). By one-pot synthesis, thiol-modified TAT-12 LCs were used as the ligands to prepare fluorescent gold nanoclusters (AuNCs@TAT-12) according to the Au-S bond between AuNCs and TAT-12. The spectra of UV-vis absorption and X-ray photoelectron spectroscopy (XPS) of AuNCs@TAT-12 indicated that the core of gold of AuNCs@TAT-12 exhibited high gold oxidation states. The fluorescence of AuNCs@TAT-12 was observed with a maximum intensity at ~352 nm coming from TAT-12 on AuNCs@TAT-12 and the fluorescence quantum yield of AuNCs@TAT-12 was calculated to be 10.1%. Furthermore, the fluorescence with a maximum intensity at ~448 nm was attributed to a ligand–metal charge transfer between the ligands of TAT-12 LCs and the core of AuNCs. The image of transmission electron microscopy (TEM) further demonstrated an approximately spherical shape of AuNCs@TAT-12 with an average size of 2.3 nm. A combination of UV-vis absorption spectra, XPS spectra, fluorescence spectra and TEM image, fluorescent AuNCs@TAT-12 were successfully synthesized via one-pot synthesis. Our work provides a practical approach to the synthesis of LCs conjugated AuNCs for future applications in nanobiotechnology.

## 1. Introduction

In recent decades, nanobiotechnology has revolutionized the biomedical field, utilizing materials whose sizes are nanometer scale. These nanomaterials exhibit unique chemical and physical characteristics more favorable to various applications, including in the biomedical field [1,2,3]. Among them, gold nanomaterials arouse particular interest in biomedical fields because of their unique optical properties and easy surface functionalization [4,5,6]. Recently, gold nanoclusters (AuNCs) composed of several to a hundred gold atoms found applications in therapy, detection, and imaging [7,8,9,10]. Moreover, AuNCs can be functionalized with organic molecules to enhance their biological properties. These molecules can be small molecules (<900 Daltons) including glucose, mercaptohexanoic acid, glutathione and quaternary ammonium or macromolecules (>900 Daltons) including transferrin, bovine serum albumin and lysozyme [11,12,13].

The nanomaterials conjugated with liquid crystals (LCs) have revealed a great potential to combine their optical and electrical properties. Different LCs can be derived from hardcore groups of some crystal compounds, such as binaphthyl derivatives [14] and triazatruxene-based derivatives [15]. The 4-cyano-4′-pentylbiphenyl, also named 5CB, is one of nematic LCs. The cyano biphenyl group is the hardcore group of the commonly used LCs of 5CB. Therefore, multiple cyano biphenyl-based LCs can be synthesized by changing the carbon number of the long chain or replacing the carbon atom on the chain with heteroatoms. With the changes in the carbon number of long chain and the carbon atom on the chain, the molecular melting point and liquid crystal phase of LCs will be changed. Furthermore, through previous studies, the nanomaterials conjugated with LCs were developed to enhance their optical and electrical properties [16,17,18]. Recent achievements have demonstrated that nematic LCs conjugated with gold nanoparticles (AuNPs) exhibit interesting optical and electrical properties [19,20,21]. For example, plasmonic AuNPs in the nematic liquid crystal 5CB exhibited an 8 nm shift in the surface plasmon resonance, and their repeated temperature cycling between the isotropic and nematic phases has also been demonstrated [22]. An acetylcholinesterase LC biosensor has been utilized for amplified detection of acetylcholine and acetylcholinesterase inhibitor based on the large size of AuNPs to disrupt the orientational arrangement of LCs [23]. A visualized LC-based system using protein-coated AuNPs was applied for imaging the disruption of a phospholipid monolayer [24]. The biological applications of LC-conjugated nanomaterials have become an interesting area for applied and basic research. However, there is still lack of design and investigation of LCs conjugated with AuNCs.

The various ligands with thiol groups have been extensively utilized to prepare AuNCs such as amino acids, peptides, antibodies and enzymes. For preparation of LC-conjugated AuNCs, the ligand of LCs must be modified with thiol groups. In this work, the hardcore of a 4-cyano biphenyl group was applied to synthesize a LC of 4′-(2-mercaptoethyl)-(1,1′-biphenyl)-4-carbonitrile (TAT-12) based on Suzuki coupling and Appel reaction. LCs of TAT-12 were measured by nuclear magnetic resonance (NMR) spectroscopy, ultraviolet-visible (UV-vis) spectroscopy and differential scanning calorimetry (DSC). Afterwards, TAT-12 with a thiol group was used as a ligand to prepare gold nanoclusters (AuNCs@TAT-12). AuNCs@TAT-12 were characterized using UV-vis spectroscopy, fluorescence spectroscopy and transmission electron microscopy (TEM).

## 2. Materials and Methods

### 2.1. Chemicals

Bis(pinacolato) diboron (≥98%), Pd(dppf) Cl_2_•CH_2_Cl_2_, and 4-bromobenzonitrile (≥97%) were purchased from Matrix Scientific (Columbia, SC, USA). 1,4-Dioxane (≥99.0%) was purchase from J.T Baker (Phillipsburg, NJ, USA). 2-(4-Bromophenyl)ethanol (98%), K_2_CO_3_ (99.0%), triphenylphosphine (99.0%) and thiourea (99.0%) were purchased from Alfa Aesar (Haverhill, MA, USA). MgSO_4_ (97%), ethyl acetate (99%), Pd (PPh_3_)_4_ (99%) and tetrabromomethane (98%) were purchased from Acros Organics (Morris, NJ, USA). Hexanes (99%) was purchase from Macron Fine Chemicals (Phillipsburg, NJ, USA). Toluene (99.8%) and tetrachloroauric (III) acid (99.9%) were purchased from Sigma-Aldrich (St. Louis, MO, USA). Dichloromethane (99+%) was purchased from DUKSAN (Ansan-si, Korea). Sodium hydroxide (NaOH, ≥98.0%) was purchased from Fluka (St. Gallen, Swiss).

### 2.2. Synthesis of 4-(4,4,5,5-Tetramethyl-1,3,2-Dioxaborolan-2-yl) Benzeneethanol (TAT-1)

In a nitrogen atmosphere, a mixture of bis(pinacolato) diboron (3.04 g, 11.9 mmol) and Pd(dppf) Cl_2_•CH_2_Cl_2_ (406 mg, 0.497 mmol) in 1,4-dioxane was mixed in a 50-mL, two-necked, round-bottom flask. The reaction mixture was degassed with nitrogen for 30 min, and 2-(4-bromophenyl)ethanol (2.00 g, 10 mmol) was added to the flask by syringe, then the reaction mixture was warmed up to 100 °C 24 h. On completion, the reaction mixture was cooled to room temperature and filtered over a pad of celite and washed with ethyl acetate. Then, the filtrate was extracted with ethyl acetate and DI water. The organic phase was dried over MgSO_4_ and concentrated by rotary evaporation. The residue was purified by column chromatography on silica gel with hexanes: ethyl acetate (30–70% ethyl acetate, by volume) as the eluent. The compound TAT-1 was obtained as a yellow solid. The ^1^H and ^13^C NMR data were the same as the reference [25].

### 2.3. Synthesis of 4′-(2-Hydroxyethyl)-(1,1′-Biphenyl)-4-Carbonitrile (TAT-3)

In a nitrogen atmosphere, a mixture of 4-bromobenzonitrile (176 mg, 0.967 mmol), TAT-1 (200 mg, 0.806 mmol), Pd (PPh_3_)_4_ (46.6 mg, 40.3 μmol) and K_2_CO_3_ (667 mg, 4.84 mmol) in 4.8 mL toluene, 4.8 mL ethanol and 2.4 mL DI water were mixed in a 25-mL, two-necked, round-bottom flask. The reaction mixture was degassed with nitrogen for 15 min, and warmed up to reflux for 2.5 h. On completion, the reaction mixture was cooled to room temperature and then extracted twice with ethyl acetate and DI water. The organic phase was dried over MgSO_4_ and concentrated by rotary evaporation. The residue was purified by column chromatography on silica gel with hexanes: ethyl acetate (20–50% ethyl acetate, by volume) as the eluent. Compound TAT-3 was obtained as a white solid (88.9% yield).

### 2.4. Synthesis of 4′-(2-Bromoethyl)-(1,1′-Biphenyl)-4-Carbonitrile (TAT-4)

In a nitrogen atmosphere, a mixture of tetrabromomethane (297 mg, 0.896 mmol) and TAT-3 (100 mg, 0.448 mmol) in 7.5 mL dichloromethane were mixed in a 25-mL, two-necked, round-bottom flask. After cooling the reaction mixture to 0 °C, triphenylphosphine (235 mg, 0.896 mmol) was added, warmed up to room temperature, and stirred 1 h. On completion, the reaction mixture was concentrated by rotary evaporation. The crude was purified by column chromatography on silica gel with hexanes: dichloromethane (50% dichloromethane, by volume) as the eluent. Compound TAT-4 was obtained as a white solid (89.8% yield).

### 2.5. Synthesis of 4′-(2-Mercaptoethyl)-(1,1′-Biphenyl)-4-Carbonitrile (TAT-12)

In a nitrogen atmosphere, a mixture of thiourea (133 mg, 1.75 mmol) and TAT-4 (250 mg, 0.874 mmol) in 1.5 mL ethanol was mixed in a 10-mL, two-necked, round-bottom flask with a condenser. The reaction mixture was warmed up to reflux for 2 h. On completion, the reaction mixture was concentrated by rotary evaporation and washed by ethyl acetate. The cleaned solids were added to the solution of NaOH (112 mg, 2.80 mmol) in DI water (1.75 mL) and rapidly stirred for 22 h. When the reaction finished, concentrated HCl was added followed by rapid stirring and then extraction with ether. The organic phase was dried over MgSO_4_ and concentrated by rotary evaporation to obtain TAT-12. Compound TAT-12 was obtained as a white solid (87.6% yield) with molecular weight of 239 g per mole (g/mol).

### 2.6. Synthesis of Gold Nanoclusters Conjugated with TAT-12

The involved materials were tetrachloroauric (III) acid (HAuCl_4_) trihydrate (ACS reagent) and TAT-12 powder. AuNCs@TAT-12 were synthesized by the one-pot synthesis. The powder of TAT-12 LCs was solubilized in deionize water via sonication for 40 min at 10 °C. Afterward, 0.5 mM of HAuCl_4_ aqueous solution was prepared. For the one-pot synthesis, equivalent volumes of TAT-12 LCs (10 mM) and HAuCl_4_ aqueous solution (0.5 mM) were mixed and then the mixture was stirred at 280 rpm and 45 °C for 24 h. After 24 h, the solution of AuNCs@TAT-12 was obtained. For further purification, the solution of AuNCs@TAT-12 was centrifuged and then the purified AuNCs@TAT-12 in the supernatant were obtained for the following experiments.

## 3. Results and Discussion

### 3.1. Synthetic Pathway of 4′-(2-Mercaptoethyl)-(1,1′-Biphenyl)-4-Carbonitrile (TAT-12)

**Figure 1:**
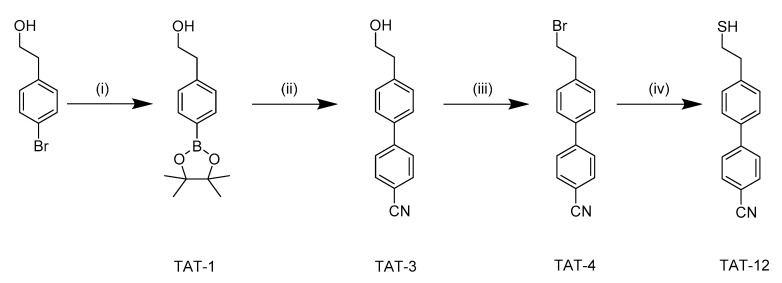
Schematic illustration of synthetic pathway for TAT-12. (**i**) bis(pinacolato) diboron, Pd(dppf)Cl_2_.CH_2_Cl_2_, KOAc, 1,4-dioxane, 100 °C. (**ii**) Pd(PPh_3_)_4_, 4-Bromobenzonitrile, K_2_CO_3_, toluene, EtOH, DI water, reflux. (**iii**) CBr_4_, PPh_3_, THF, 0 °C to room temperature (**iv**) (1) thiourea, EtOH, reflux (2) 1.6 M NaOH, stir r.t, then add concentrated HCl.

Treatment of 2- (4-bromophenyl) ethanol with borate was used to obtain TAT-1. After that, TAT-1 acted in the presence of Pd-catalysts, followed by 4-bromobenzonitrile to produce TAT-3 by Suzuki coupling. Finally, the Appel reaction was used to replace the hydroxyl group to the terminal bromide group followed by thiourea to result in TAT-12. Detailed synthesis methods are shown in Figure 1.

### 3.2. Characterizations of TAT-12

**Figure 2:**
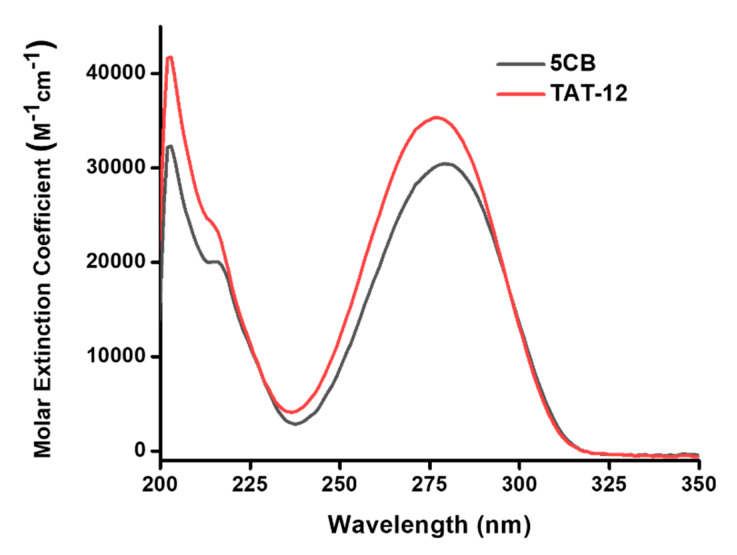
UV-Vis spectra of TAT-12 and 5CB in MeOH (10^−5^ M).

To explore the properties of TAT-12 with thiol groups, nuclear magnetic resonance (NMR) spectroscopy, ultraviolet-visible (UV-vis) spectroscopy and differential scanning calorimetry (DSC) were applied to characterize the structural and optical properties of TAT-12. To characterize structural property, TAT-3, TAT-4 and TAT-12, respectively, were dissolved in deuterochloroform (CDCl_3_) for ^1^H and ^13^C NMR measurements. The ^1^H NMR and ^13^C NMR spectra of TAT-3, TAT-4 and TAT-12 indicated the successful syntheses of TAT-3, TAT-4 and TAT-12, as shown in Figures S1–S3 in the supporting information. To study the liquid crystal properties of TAT-12, differential scanning calorimetry (DSC) was used to measure the phase transition temperature. For the experimental conditions of DSC, the heating rate was 10 °C/min, and the scanning was repeated three times. As shown in Figure S4, the melting point of TAT-12 was located at about 92 °C. After cooling of TAT-12, two absorption peaks were observed at 63 and 61 °C, indicating liquid crystal properties. To investigate the optical properties, TAT-12 and 5CB were dissolved in MeOH for UV-vis measurements. As shown in Figure 2, the molecular absorptions of TAT-12 and 5CB are mainly distributed from 200 to 325 nm. The maximum absorption of 5CB was located at ~275 nm due to the π→π* transition mode. Compared to 5CB, the UV-vis spectra of TAT-12 with the same hardcore of 5CB only revealed a slight blue shift (~3 nm). The result of the slight blue shift in UV-vis spectra indicated that the replacement of alkylation on 5CB core to the thiol group has shown a slight effect on the aromatic conjugation for TAT-12. Therefore, TAT-12 was supposed to reveal liquid crystal properties.

### 3.3. Optical and Structural Properties of AuNCs@TAT-12

**Figure 3:**
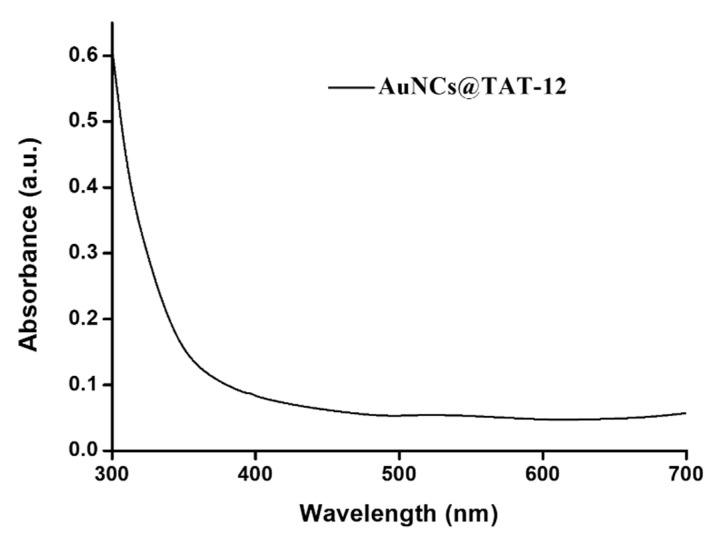
UV−vis absorption spectrum of AuNCs@TAT-12.

To investigate the optical and structural properties, AuNCs@TAT-12 were characterized using UV-vis spectroscopy (JASCO V-770 Spectrophotometer, Easton, MD, USA), fluorescence spectroscopy (JASCO spectrofluorometer FP-8500) and transmission electron microscopy (TEM) (HITACHI HT7700, Tokyo, Japan). The optical properties of AuNCs@TAT-12 were highlighted through UV-vis and fluorescence spectra. The UV-vis absorption spectra of AuNCs@TAT-12 revealed no significant absorption at ~520 nm because AuNCs@TAT-12 does not exhibit a surface plasmon resonance, as shown in Figure 3. The reason for the disappeared surface plasmon absorption of AuNCs@TAT-12 can be attributed to the core of gold of AuNCs@TAT-12 revealing a high gold oxidation states [10,26,27].

**Figure 4:**
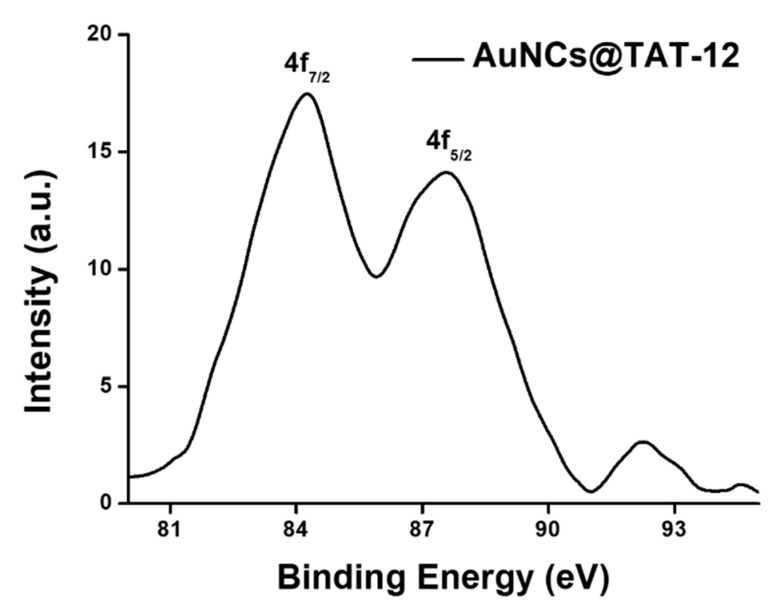
XPS spectra of AuNCs@TAT-12 Cys-AuNCs. The peaks of Au 4f_5/2_ and Au 4f_7/2_ were located at 87.6 and 84.2 eV, respectively.

To examine the core of gold of AuNCs@TAT-12, which revealed high gold oxidation states, XPS spectra were applied to measure the binding energy of gold for AuNCs@TAT-12. In XPS spectra of Figure 4, the peaks in Au 4f_5/2_ and Au 4f_7/2_ for AuNCs@TAT-12 were located at 87.8 and 84.2 eV, respectively. For the bulk gold, the peaks in Au 4f_5/2_ and Au 4f_7/2_ were located at 87.4 and 84.0 eV, respectively. For Au 4f, XPS spectra of AuNCs@TAT-12 indicated a positive shift compared to that of the bulk gold because AuNCs@TAT-12 contained lots of Au^+1^ [28]. The results of XPS measurements corresponded to the results of the analysis of UV-vis spectra.

**Figure 5:**
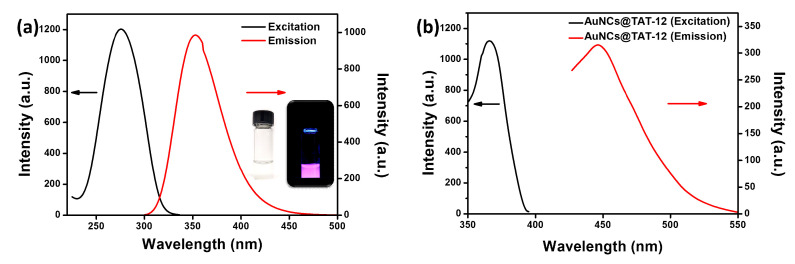
(**a**) Excitation spectrum of AuNCs@TAT-12 with fluorescence wavelength at 352 nm. Emission spectrum of AuNCs@TAT-12 under excitation wavelength of 273 nm. (**b**) Excitation spectrum of AuNCs@TAT-12 with fluorescence wavelength at 448 nm. Emission spectrum of AuNCs@TAT-12 under excitation wavelength of 366 nm. In Figure 5a, the insets showed the photograph of AuNCs@TAT-12 under white light (left) and fluorescence image of AuNCs@TAT-12 irradiated by handheld UV lamp (right).

To further study the optical properties, fluorescence spectra of AuNCs@TAT-12 were examined. As shown in Figure 5a, under an excitation wavelength of 273 nm, the emission spectrum of AuNCs@TAT-12 revealed a maximum intensity at ~352 nm. Compared to fluorescence spectrum of TAT-12, the fluorescecne with a maximum intensity at ~352 nm was attributed to the fluorescecne coming from TAT-12 on AuNCs@TAT-12. The fluorescence quantum yield of TAT-12 on AuNCs was calculated to be 10.1% by integrating the sphere (JASCO ILF-835). Furthermore, there is an excitation peak located at wavelength of ~366 nm, as shown in Figure 5b. Under excitation wavelength of 366 nm, the emission spectrum of AuNCs@TAT-12 revealed a maximum intensity at ~448 nm due to the ligand–metal charge transfer between the thiolate ligand of TAT-12 and the core of AuNCs. Previous studies have demonstrated that the fluorescence of AuNCs can be attributed to ligand–metal charge transfer between the thiolate ligand and the core of AuNCs. With the ligand–metal charge transfer, the electrons were delivered from sulfur atoms of TAT-12 ligand to the core of AuNCs. The results of fluorescence properties indicated that the ligand of TAT-12 was successfully conjugated with AuNCs to obtain AuNCs@TAT-12 based on the Au-S bond. The insets in Figure 5a showed the photograph of AuNCs@TAT-12 under white light (left) and fluorescence image of AuNCs@TAT-12 irradiated by handheld UV lamp (right). The red-violet fluorescence of AuNCs@TAT-12 can be clearly observed with irradiation by handheld UV lamp.

**Figure 6:**
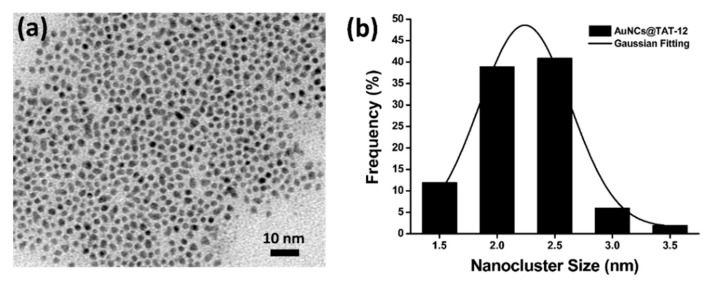
(**a**) TEM image of AuNCs@TAT-12 and (**b**) a histogram of nanocluster size distribution of AuNCs@TAT-12 and its Gaussian fitting curve.

The structure of AuNCs@TAT-12 was characterized by TEM. In Figure 6a, AuNCs@TAT-12 were distinctly separated because AuNCs@TAT-12 were protected by an organic compound of TAT-12 LCs to avoid the aggregation of nanoclusters. The TEM image also revealed an approximately spherical shape of AuNCs@TAT-12. The average size of AuNCs@TAT-12 was calculated according to 100 nanoclusters in Figure 6a. In a histogram of Figure 6b, the size distribution of AuNCs@TAT-12 and its Gaussian fitting curve were calculated. The average size of AuNCs@TAT-12 was calculated to be 2.3 nm according to the histogram. Overall, UV-vis absorption spectra, fluorescence spectra and TEM image consolidated the idea that fluorescent AuNCs@TAT-12 were successfully synthesized via the one-pot method.

## 4. Conclusions

In conclusion, TAT-12 based on commonly used LCs of 5CB hardcore was synthesized via Suzuki coupling and Appel reaction. ^1^H NMR and ^13^C NMR spectra demonstrated the successful synthesis of TAT-12 LCs with a thiol group. The optical properties of TAT-12 showed a slight blue shift of 3 nm in comparison with 5CB LCs. DSC measurements revealed the liquid crystal properties of TAT-12 with a melting point at 92 °C and crystallization points at 63 and 61 °C. The LC of TAT-12 was applied as a ligand to synthesize AuNCs@TAT-12 by one-pot synthesis. The fluorescence of AuNCs@TAT-12 was measured with the maximum intensity at ~352 nm and the fluorescence quantum yield of 10.1%. According to TEM image, AuNCs@TAT-12 exhibited an approximately spherical shape with an average size of 2.3 nm. Overall, AuNCs@TAT-12 could be applied in the photoswitching applications in the near future due to their unique structural and optical properties.

## Supplementary Materials

The following are available online at https://www.mdpi.com/2079-4991/10/9/1755/s1, Figure S1: ^1^H NMR (upper) and ^13^C NMR (lower) spectra of TAT-3 in CDCl_3_; Figure S2: ^1^H NMR (upper) and ^13^C NMR (lower) spectra of TAT-4 in CDCl_3_; Figure S3: ^1^H NMR (upper) and ^13^C NMR (lower) spectra of TAT-12 in CDCl_3_; Figure S4: The DSC plots of TAT-12.
