# Supplementary material for: One-Pot Synthesis of Thiol-Modified Liquid Crystals Conjugated Fluorescent Gold Nanoclusters
Source: Nanomaterials (Basel). 2020 Sep 6;10(9):1755. doi: 10.3390/nano10091755 (PMC7558681; doi:10.3390/nano10091755)
Supplement: Supplementary file 1 [file nanomaterials-10-01755-s001.pdf]

Supporting Information

One-Pot Synthesis of Thiol-modified Liquid Crystals Conjugated Fluorescent Gold Nanoclusters

Po-Hsuan Hsu [1,†], Sibidou Yougbaré [2,3,†], Jui-Chi Kuo [1,†],Dyah Ika Krisnawati [4], Achmad Jazidie [5,6], Mohammad Nuh [7], Po-Ting Chou [8], Yu-Cheng Hsiao [2,8,*] and Tsung-Rong Kuo[2,9,*]

[1] School of Biomedical Engineering, College of Biomedical Engineering, Taipei Medical University, Taipei 11031, Taiwan; b812106028@tmu.edu.tw (P.-H.S.); b812105002@tmu.edu.tw (J.-C.K.)
[2] International Ph.D. Program in Biomedical Engineering, College of Biomedical Engineering, Taipei Medical University, Taipei 11031, Taiwan; d845107003@tmu.edu.tw (S.Y.)
[3] Institut de Recherche en Sciences de la Santé (IRSS-DRCO)/Nanoro, 03 B.P 7192, Ouagadougou 03, Burkina Faso
[4] Dharma Husada Nursing Academy, Kediri, East Java 64114, Indonesia; dyahkrisna77@gmail.com (D.I.K.)
[5] Department of Electrical Engineering, Institut Teknologi Sepuluh Nopember, Surabaya 60111, Indonesia; rektor@unusa.ac.id
[6] Universitas Nahdlatul Ulama Surabaya, Surabaya 60111, Indonesia
[7] Department of Biomedical Engineering, Institut Teknologi Sepuluh Nopember, Surabaya 60111, Indonesia; nuh@ee.its.ac.id or nuh@unusa.ac.id
[8] Graduate Institute of Biomedical Optomechatronics, Taipei Medical University, Taipei 11031, Taiwan; d97223108@ntu.edu.tw; ychsiao@tmu.edu.tw (Y.-C.H.)
[9] Graduate Institute of Nanomedicine and Medical Engineering, College of Biomedical Engineering, Taipei Medical University, Taipei 11031, Taiwan; trkuo@tmu.edu.tw
* Correspondence: ychsiao@tmu.edu.tw (Y.-C.H.); trkuo@tmu.edu.tw (T.-R.K.)
† These authors contributed equally to this work.

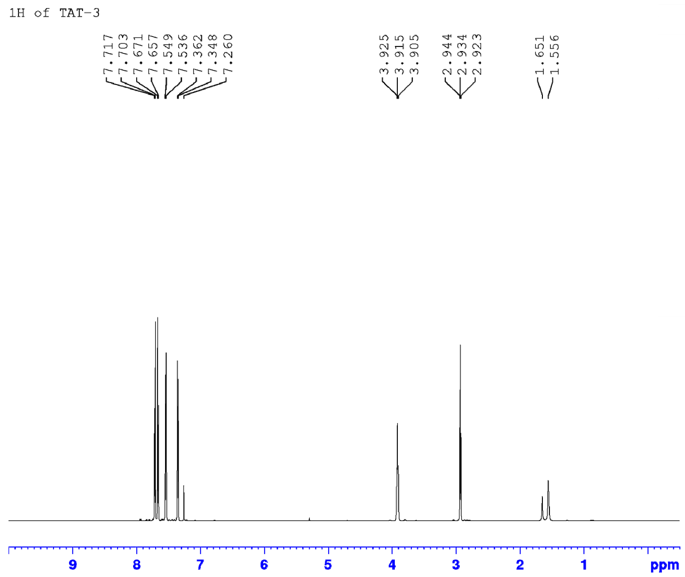

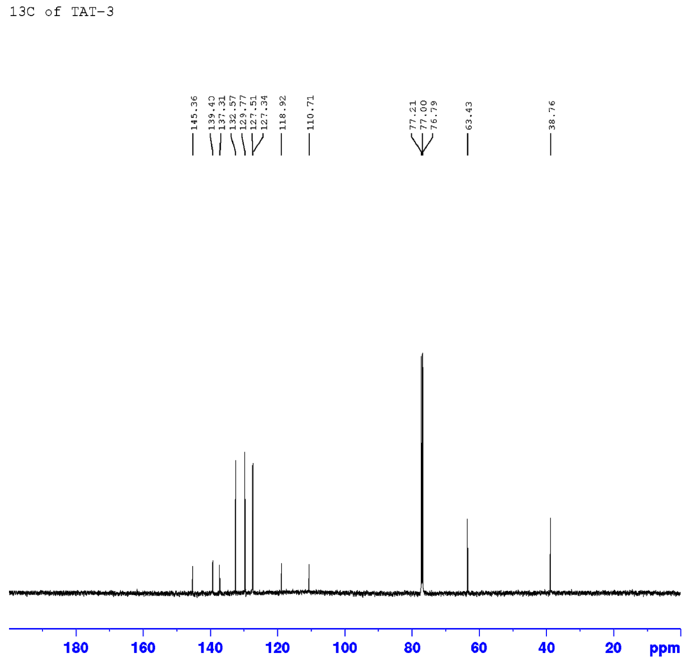

Figure S1. ^{1}H NMR (upper) and ^{13}C NMR (lower) spectra of TAT-3 in $CDCl_3$. ^{1}H NMR (600 MHz, $CDCl_3$): δ (ppm) = 7.71 (*d*, *J* = 8.2 Hz, 2H), 7.66 (*d*, *J* = 8.2 Hz, 2H), 7.54 (*d*, *J* = 8.0 Hz, 2H), 7.35 (*d*, *J* = 8.0 Hz, 2H), 3.92 (*t*, *J* = 5.9 Hz, 2H), 2.93 (*t*, *J* = 6.5 Hz, 2H); ^{13}C NMR (150 MHz, $CDCl_3$): δ (ppm) =145.36, 139.40, 137.31, 132.57, 129.77, 127.51, 127.34, 118.92, 110.71, 63.43, 38.76; HRMS (EI) *m*/*z*: $[M]^+$ calcd for $C_{15}H_{13}NO$: 223.0997, found 223.0993.

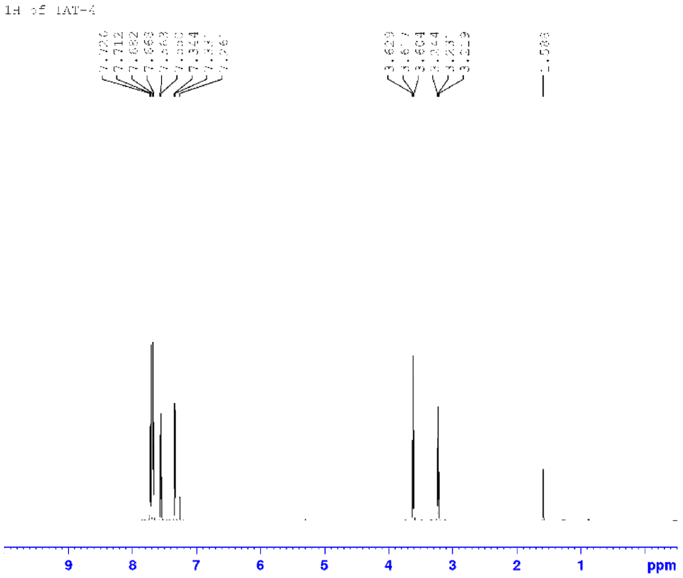

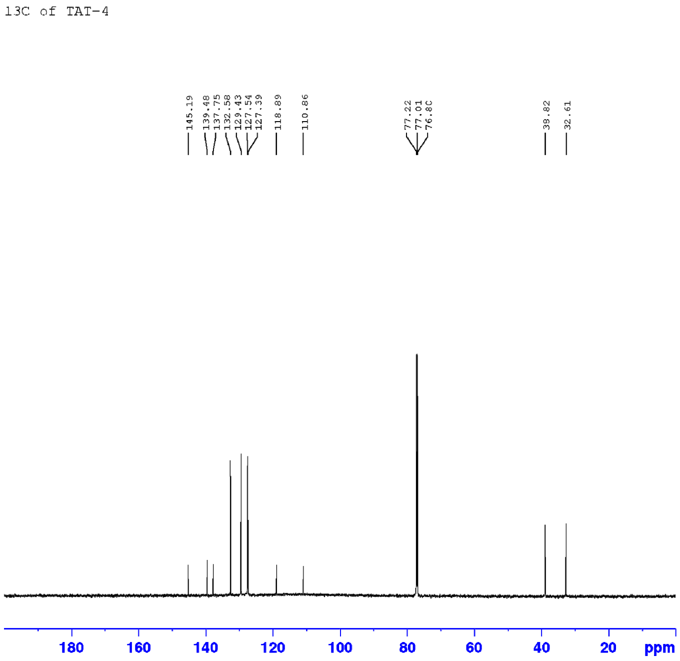

Figure S2. ^{1}H NMR (upper) and ^{13}C NMR (lower) spectra of TAT-4 in $CDCl_3$. ^{1}H NMR (600 MHz, $CDCl_3$): δ (ppm) = 7.72 (*d*, *J* = 8.1 Hz, 2H), 7.68 (*d*, *J* = 8.2 Hz, 2H), 7.56 (*d*, *J* = 8.0 Hz, 2H), 7.34 (*d*, *J* = 8.0 Hz, 2H), 3.62 (*t*, *J* = 7.4 Hz, 2H), 3.23 (*t*, *J* = 7.4 Hz, 2H); ^{13}C NMR (150 MHz, $CDCl_3$): δ (ppm) =145.19, 139.48, 137.75, 132.58, 129.43, 127.54, 127.39, 118.89, 110.86, 38.82, 32.61.

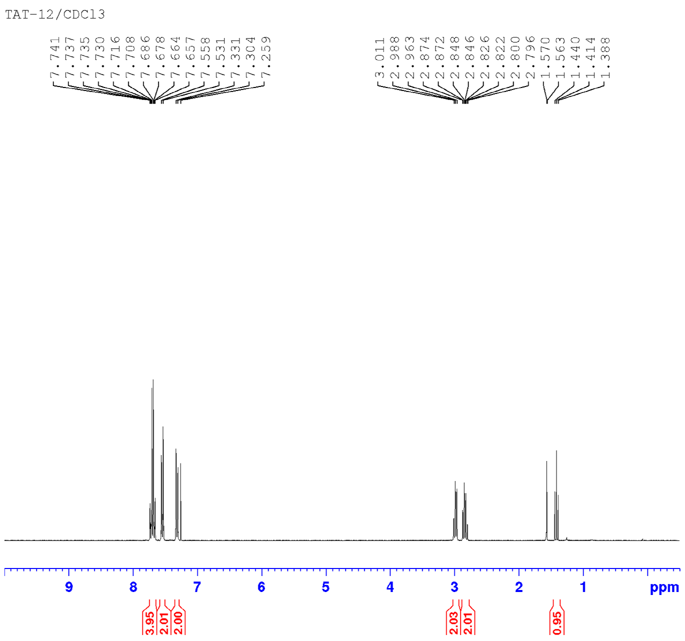

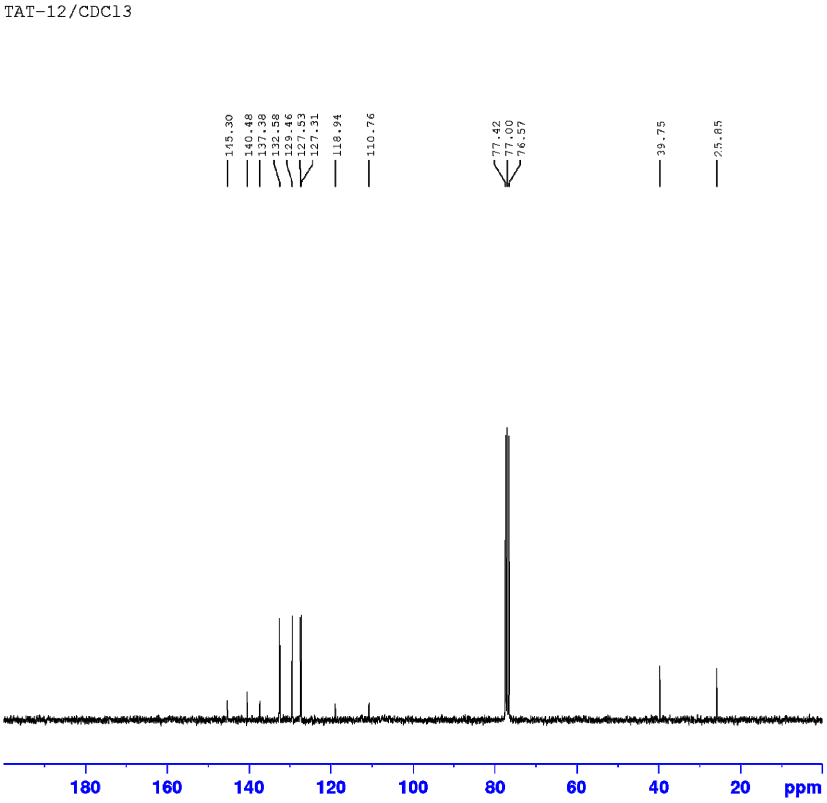

Figure S3. ^{1}H NMR (upper) and ^{13}C NMR (lower) spectra of TAT-12 in $CDCl_3$. ^{1}H NMR (300 MHz, $CDCl_3$): δ (ppm) = 7.74–7.66 (m, 4H), 7.54 (*d*, *J* = 8.2 Hz, 2H), 7.32 (*d*, *J* = 8.2 Hz, 2H), 2.99 (*t*, *J* = 7.1 Hz, 2H), 2.87–2.80 (m, 2H), 1.41 (*t*, *J* = 7.8 Hz, 2H); ^{13}C NMR (75 MHz, $CDCl_3$): δ (ppm) = 145.30, 140.48, 137.38, 132.58, 129.46, 127.53, 127.31, 118.94, 110.76, 39.75, 25.85; HRMS (EI) m/z: $[M]^+$ calcd for $C_{15}H_{13}NS$: 239.0769, found 239.0769.

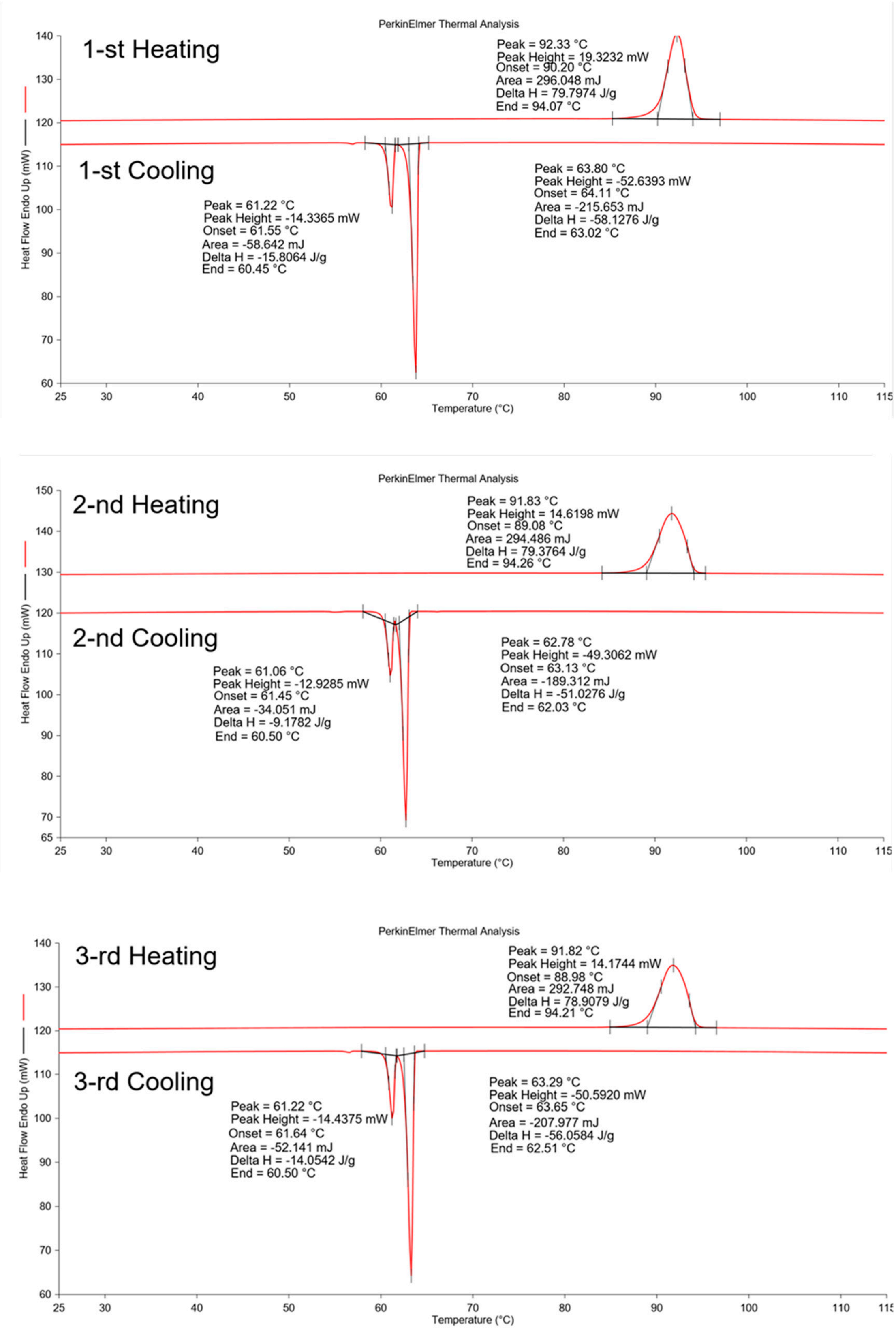

Figure S4. The DSC plots of TAT-12.
